# Cost-effectiveness of Interferon-free therapy for Hepatitis C in Germany - an application of the efficiency frontier approach

**DOI:** 10.1186/s12879-015-1048-z

**Published:** 2015-07-30

**Authors:** Christian Gissel, Georg Götz, Jörg Mahlich, Holger Repp

**Affiliations:** Department of Economics and Business, Justus Liebig University Giessen, Giessen, Germany; General Medicine, Department of Medicine, Justus Liebig University Giessen, Klinikstrasse 32, 35392 Giessen, Germany; Janssen Pharmaceutical K.K., 5-2, Nishi-kanda 3-chome, Chiyoda-ku, Tokyo 101-0065 Japan; Düsseldorf Institute for Competition Economics (DICE), University of Düsseldorf, Düsseldorf, Germany

**Keywords:** Hepatitis C, Interferon-free therapy, Sofosbuvir, Simeprevir, Cost-effectiveness, Germany, Efficiency frontier

## Abstract

**Background:**

The approval of direct-acting antivirals for Interferon-free treatment revolutionized the therapy of chronic Hepatitis C infection. As of August 2014, two treatment regimens for genotype 1 infection received conditional approval in the European Union: Sofosbuvir and Ribavirin for 24 weeks and Sofosbuvir and Simeprevir with or without Ribavirin for 12 weeks. We aim to analyze the cost-effectiveness of both regimens in Germany.

**Methods:**

We set up a Markov model with a lifetime horizon to simulate immediate treatment success and long-term disease progression for treatment-naive patients. The model analyzes both short-term and long-term costs and benefits from the perspective of the German Statutory Health Insurance. We apply the efficiency frontier method, which was suggested by German Institute for Quality and Efficiency in Health Care for cost-effectiveness analysis in Germany.

**Results:**

The efficiency frontier is defined by dual therapy and first generation direct-acting antiviral Boceprevir, yielding a maximum of € 1,447.69 per additional percentage point of sustained virologic response gained. Even without rebates, Sofosbuvir/Simeprevir is very close with € 1,560.13 per additional percentage point. It is both more effective and less expensive than Sofosbuvir/Ribavirin.

**Conclusions:**

In addition to higher sustained virologic response rates, new direct-acting antivirals save long-term costs by preventing complications such as liver cirrhosis, hepatocellular carcinoma and ultimately liver transplants, thereby offsetting part of higher drug costs. Our findings are in line with the guidance published by German Society for Gastroenterology, Digestive and Metabolic Diseases, which recommends Sofosbuvir/Simeprevir for Interferon ineligible or intolerant patients.

## Background

Hepatitis C is one of the most common chronic infectious diseases worldwide and in Germany. The total number of patients infected with hepatitis C virus (HCV) is declining in Germany, with an estimated 4,980 new infections in 2013 [1]. In contrast to the declining number of patients, severe long-term complications such as decompensated cirrhosis (DCC), hepatocellular carcinoma (HCC) and ultimately liver transplants (LT) are estimated to be increasing due to the ageing of the existing infected population. Of the HCV-infected patients in Germany, 62 % are infected with HCV genotype 1, which is most difficult to treat [2].

Recently approved direct-acting antiviral agents (DAA) have improved sustained virologic response (SVR) rates compared to the classic combination therapy of PegInterferon (IFN) and Ribavirin (RBV; combination with IFN: PR). Despite higher SVR rates close to 100 %, high treatment costs have stirred a public debate on the pricing of recently approved DAAs [3, 4].

While triple therapy with first generation protease inhibitors (PI) Boceprevir or Telaprevir improved SVR rates compared to PR, IFN was still required with both agents. One of the greatest advantages of new DAAs is the advent of IFN-free treatment regimens. IFN-free therapies provide dramatically increased SVR rates compared to first-generation DAAs and avoid IFN’s side effects like depression, anxiety and fatigue, which in many cases has negative effects on patients’ ability to work [5]. Another advantage of recently approved DAAs are shorter treatment durations, which can contribute to a better compliance.

As of August 2014, two treatment regimens are recommended for IFN-free therapy of HCV genotype 1 infection by German Society for Gastroenterology, Digestive and Metabolic Diseases (DGVS) guidelines [6]:

- Sofosbuvir (SOF) and Simeprevir (SMV) combination therapy with or without RBV for 12 weeks
- SOF and RBV combination therapy for 24 weeks

Since the approval of DAAs, HCV therapy has played a major economic role in the German Statutory Health Insurance (SHI) funds. In 2013, the German SHI funds spent € 48.6 mn on Telaprevir and € 19.0 mn on Boceprevir [7].

In 2011, Germany introduced early benefit assessments and rebate negotiations as a measure of regulating pharmaceutical reimbursement [8]. After approval for the German market, pharmaceutical manufacturers are free to set a price for one year. Within that one year after approval, all prices of newly approved drugs need to be negotiated with the German SHI funds. The outcome of the negotiations is a rebate agreement, which comes into effect one year after approval in Germany [9].

Negotiations for the rebate agreement are based on a 6-month early benefit assessment, which determines additional benefits of the new therapy compared to an existing alternative therapy [10]. Early benefit assessments are conducted by German Federal Joint Committee (GBA). GBA’s assessment in turn relies on a 3-month assessment by German Institute for Quality and Efficiency in Health Care (IQWIG).

The outcomes of cost-effectiveness analyses with the efficiency frontier approach are maximum reimbursable prices. Under current German legislation, no cost-effectiveness analysis is involved in the process of the initial rebate negotiations [11]. However, cost-effectiveness analysis can be requested by either negotiating party as a base for further negotiations, once a first rebate agreement has been reached, *i.e.,* the maximum reimbursable price would serve as a base for a new rebate negotiation between manufacturer and SHI funds. We aim to analyze the cost-effectiveness of DAAs for IFN-free therapies in this context.

Cost-effectiveness analyses are conducted by IQWIG and they are based on the efficiency frontier method [12, 13]. IQWIG’s method has previously been criticized because it does not use quality-adjusted life years (QALYs) as a measure of patient benefits. Contrary to approaches based on QALYs, German IQWIG requires the definition of specific clinical parameters as a measure of patient benefits, *i.e.,* the method is not suitable to define a global threshold per QALY gained, as practiced in England and Wales [14].

Application of IQWIG’s efficiency frontier method requires the assessment of previously existing therapies. The ratio of previous increases in effectiveness and costs determines the efficiency frontier. This was heavily criticized by health economists [14, 15]. The results of efficiency frontier analyses depend on the benefits and costs of previously existing therapies, even though these therapies have never been subject to health technology assessment and no willingness-to-pay can be derived from the prices, which could previously be unilaterally determined by manufacturers.

If the ratio of previous gains in benefits and costs has been relatively expensive (*i.e.,* relatively higher costs per clinical benefit unit), the efficiency frontier approach leads to higher maximum reimbursable prices for new approvals, too. This might not be desired from a regulatory perspective. Another problem of application can arise if multiple efficiency frontiers need to be computed to reflect the relevance of more than one clinical parameter. Naturally, the results of different efficiency frontiers may vary, potentially leading to conflicting results. IQWIG suggested to aggregate specific efficiency frontier results based on patient preferences as found by analytic hierarchy processes or conjoint analyses [16–18]. We aim to analyze whether the problems outlined above prevent useful results if the efficiency frontier method is applied to DAAs for HCV in Germany.

## Methods

Both IQWIG and GBA have accepted SVR rates as the most important clinical parameter determining the success of antiviral therapy for patients suffering from chronic HCV infection. In its early benefit assessments of SOF and SMV, German Federal Joint Committee defined both classic dual therapy (PR) and triple therapy with first generation PIs (Boceprevir or Telaprevir) as appropriate comparators. Therefore, we define the efficiency frontier with PR and Boceprevir triple therapy as Boceprevir was the first PI to be approved in Germany.

We model the cost-effectiveness of the two comparators and the two IFN-free therapies with an extended Markov model, which is also used for analysis in the United Kingdom and in Japan [19, 20]. The model simulates the pathways of treatment-naive patients in all possible states of fibrosis (F0-F4). The model has a lifetime time horizon with a cycle length of one year. The cycle length is consistent with previous economic models and reflects the relatively slow progression rate for chronic HCV infection [21, 22].

All modeling characteristics were adapted to reflect clinical practice in Germany. Effectiveness analysis is based on GBA’s assessment of PR and Boceprevir triple therapy as part of early benefit assessments. However, no IFN-free therapy has been assessed by GBA yet. Therefore, benefit analysis of IFN-free regimens is only based on the recommendations published by DGVS [6]. Therefore, the model was extended to incorporate suitable studies to simulate treatment with IFN-free regimens. For SOF/SMV, the COSMOS trial was included in the model, as it reports SVR rates for treatment naive patients after 12 weeks [23]. For SOF/RBV the QUANTUM trial results were used [24].

We conducted a Pubmed search for 'hepatitis c cost germany' to adapt the model to German prices. Out of 65 hits, only 2 studies could be identified as reporting original German cost data for HCV [25, 26]. All costs used in the model are in 2014 Euros and were discounted by 3 %, as suggested by IQWIG. All drug costs reflect German prices as of August 2014 (according to Lauer-Taxe).

**Fig. 1:**
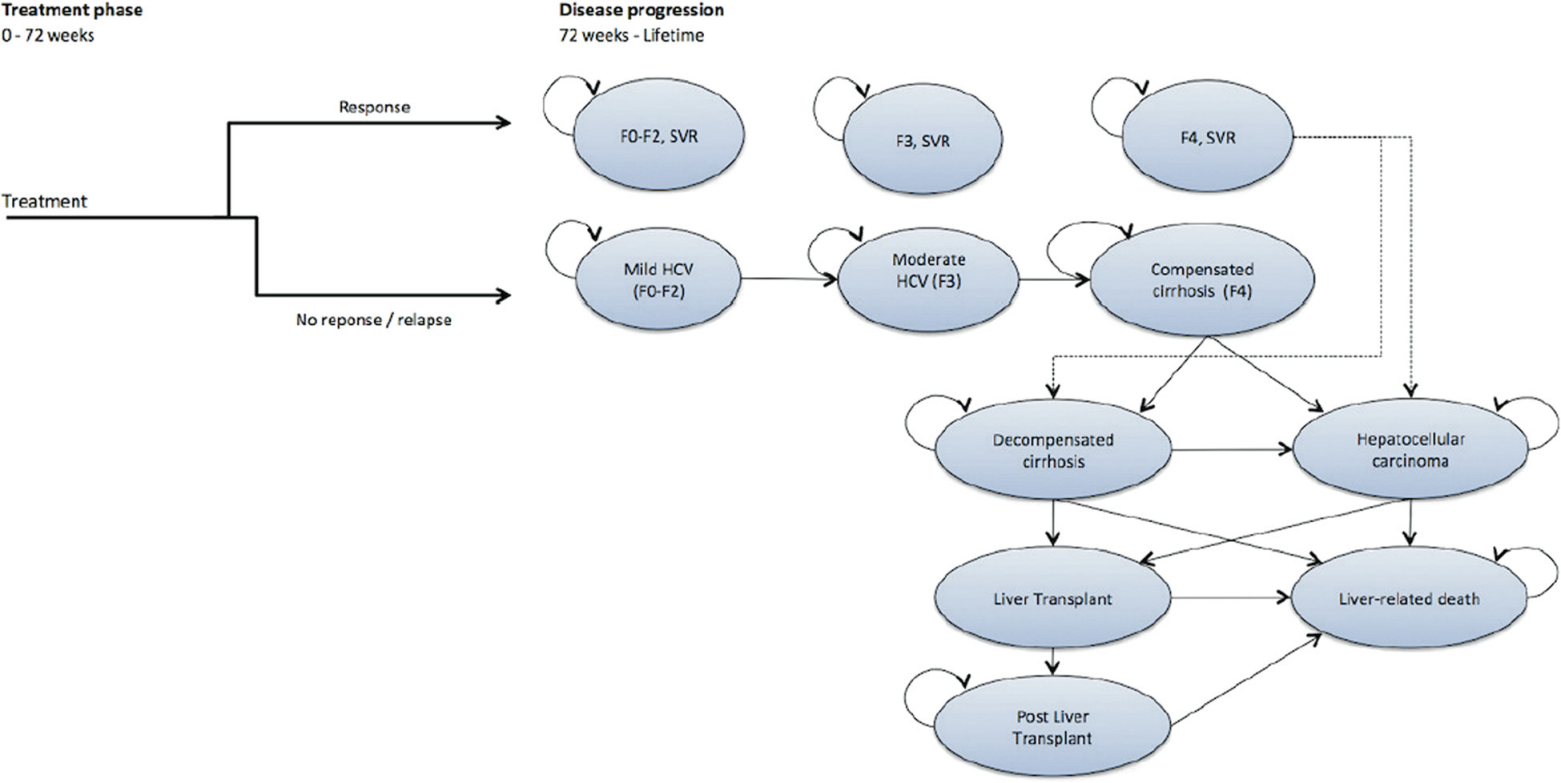
Model structure. The Markov model simulates both immediate treatment success and long-term disease progression. For simplicity, all-cause mortality is not explicitly depicted

The model analyzes two components of health economic interest: The antiviral therapy phase with a follow-up until week 72, and the disease progression with a lifetime perspective (see Fig. 1).

The second phase depends on the outcomes of the first phase. The model is not limited to the immediate treatment phase, but takes a lifetime perspective, because long-term complications associated with HCV are very important for a chronic and severe disease like HCV (Table 1). The transition probabilities of the economic model are listed in Table 2. As no specific data for the German context could be found, the model primarily relies on a recent, systematic literature review published as part of an English Health Technology Assessment, which proved to be suitable for the purposes of our model [21].

**Table 1:** Costs of adverse events and long-term complications

On-treatment adverse events	2014 costs	Source	Probabilistic Sensitivity Analysis		
			Distribution	Lower	Upper
Anaemia	1,821.25 €	[25]	Gamma	1,457.00 €	2,185.50 €
Neutropenia	42.54 €	expert opinion	Gamma	34.03 €	51.05 €
Rash	26.85 €	[25]	Gamma	21.48 €	32.22 €
Pruritus	22.58 €	[25]	Gamma	18.06 €	27.10 €
Long-term complications					
Decompensated cirrhosis	9,656.36 €	[25]	Gamma	7,725.09 €	11,587.63 €
Hepatocellular carcinoma	22,762.62 €	[26]	Gamma	18,210.10 €	27,315.14 €
First year after liver transplant	155,815.33 €	[26]	Gamma	124,652.26 €	186,978.40 €
Further years after liver transplant	22,534.99 €	[26]	Gamma	18,027.99 €	27,041.99 €

**Table 2:** Annual transition probabilities in the economic model

From	To	Mean	SE	Source	Probabilistic Sensitivity Analysis		
					Distribution	Lower	Upper
F0-F2	F3	0.025	0.004	[21, 32]	Beta	0.018	0.033
F3	F4	0.037	0.007	[21, 32]	Beta	0.025	0.052
F4	DCC	0.039	0.010	[21, 33]	Beta	0.022	0.061
	HCC	0.014	0.010	[21, 33]	Beta	0.002	0.039
SVR F4	DCC	0.000	0.000	expert opinion	Beta	0.000	0.000
	HCC	0.005	0.001	[34]	Beta	0.004	0.007
DCC	HCC	0.014	0.010	[21, 33]	Beta	0.002	0.039
	LT	0.020	0.003	[21, 35]	Beta	0.015	0.026
	LrD	0.130	0.010	[21, 33]	Beta	0.111	0.150
HCC	LT	0.020	0.003	[32, 35]	Beta	0.015	0.026
	LrD	0.430	0.030	[21, 33]	Beta	0.372	0.489
LT	LrD	0.150	0.023	[21, 36]	Beta	0.109	0.197
pLT	LrD	0.057	0.009	[21, 36]	Beta	0.041	0.075
All-cause mortality		Age/gender specific		Life Tables, German Federal Office of Statistics			

*DCC* decompensated cirrhosis, *HCC* hepatocellular carcinoma, *SVR* sustained virologic response, *LT* liver transplant, *LrD* Liver related death, *pLT* post liver transplant, fibrosis states F0-F4 according to METAVIR

The model is run to simulate the treatment success of the four therapeutic options for our efficiency frontier analysis: PR, Boceprevir + PR, SOF/RBV, SOF/SMV. The most important baseline characteristic of simulated patients is the degree of fibrosis according to the METAVIR scoring system. Each treatment is simulated for the duration as suggested by DGVS guidelines. After each treatment, patients can either have undetectable HCV-RNA and achieve SVR or fail therapy and be assigned to the relapser group. During each treatment course, on-treatment adverse events can appear. Furthermore, all-cause mortality was applied to all possible health states according to German life tables (not shown in Fig. 1 for simplicity). Detailed descriptions of the model's mechanics have been published elsewhere [19, 20].

In order to determine the influence of uncertainty surrounding input parameters, we provide a multivariate probabilistic sensitivity analysis. Parameter estimates were varied within the uncertainty distributions that best reflect the nature of each specific parameter. Uncertainty margins are applied to each input parameter of interest based on corresponding intervals provided in the literature (Table 2) or based on assumptions if information is unavailable. The standard error was assumed to vary 20 % around the mean in case information on variance was not available for a specific parameter (applied to German cost data in Table 1).

We process 3000 simulations, which vary the parameters by random draws from their assumed distributions (as shown in Tables 1 and 2), to represent the uncertainty of model results. We plot the 3000 simulations in a scatterplot in Fig. 3 and compute the percentage of simulations, in which SOF/SMV or SOF/RBV would be above or on the efficiency frontier.

## Results

As of August 2014, one week of SMV treatment costs € 3,829.91, while one week of SOF treatment costs € 4,714.69, i.e. SOF/SMV combination therapy costs € 102,535.14 for 12 weeks, while SOF/RBV costs € 113,152.56 for 24 weeks (DAA costs excluding RBV).

**Fig. 2:**
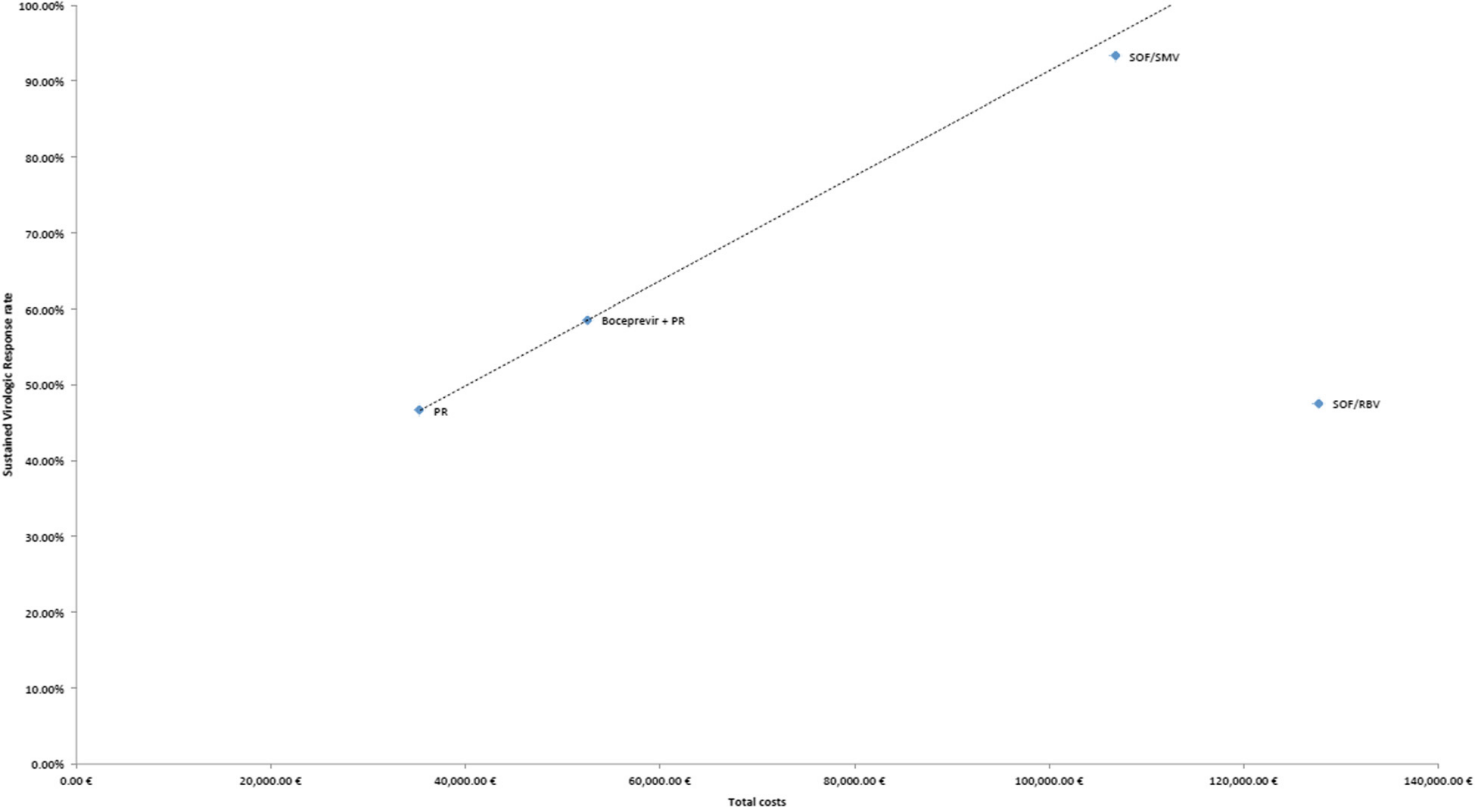
Efficiency frontier. The efficiency frontier plots the required ratio of increases in sustained virologic response rates and costs, as defined by comparators dual therapy (PR) and Boceprevir triple therapy

The main efficiency frontier for treatment-naive patients as reported in Fig. 2 is defined by PR and Boceprevir triple therapy. SVR was 46.62 % for treatment with PR for treatment-naive patients. Boceprevir triple therapy improved SVR rates to 58.58 %.

Boceprevir combination therapy increased costs from € 35,358.93 to € 52,679.70. The increase in total costs is mainly due to € 23,606.10 in PI costs. Along with a better SVR rate, long-term complications and the costs associated with them decreased. The introduction of Boceprevir defined a required marginal efficiency of a maximum of € 1,447.69 per additional SVR percentage point gained. According to IQWIG’s efficiency frontier concept, every new therapy for treating HCV can only cost an additional € 1,447.69 for each additional SVR percentage point, which is gained by the new therapy beyond the rate of 58.58 % which is achievable with Boceprevir (Fig. 2).

In our modeling approach, IFN-free regimen SOF/RBV achieves a rate of 47.37 %, which is below Boceprevir’s SVR rate for treatment-naive patients. Therefore, the required marginal efficiency of SOF/RBV cannot be computed.

Our results show that the combination of SOF/SMV is more effective and cheaper than SOF/RBV (Table 3). SOF/SMV achieves a SVR rate of 93.31 %, *i.e.,* adding 34.73 percentage points to the previously achievable results with Boceprevir. Applying the efficiency frontier cost per additional SVR percentage point, these SVR rates result in maximum costs of € 102,963.08 for SOF/SMV. In our model, SOF/SMV comes very close to this threshold, even though no rebates have been negotiated for SOF or SMV yet. Total costs are € 106,868.76 for SOF/SMV for 12 weeks, *i.e.,* € 3,905.68 or 3.8 % above the threshold defined with the efficiency frontier approach.

**Table 3:** Main modeling outcomes

Treatment naïves population				
	PR	Boceprevir + PR	SOF/RBV	SOF/SMV
Clinical outcomes				
SVR (24 weeks after tx)	46.62 %	58.58 %	47.37 %	93.31 %
Decompensated cirrhosis	11.88 %	10.07 %	10.64 %	1.73 %
Hepatocellular carcinoma	5.65 %	4.97 %	5.30 %	2.15 %
Liver Transplantation	1.42 %	1.22 %	1.27 %	0.26 %
Liver related death	13.52 %	11.71 %	12.22 %	3.22 %
Costs				
Total Costs	35,358.93 €	52,679.70 €	127,891.69 €	106,868.76 €
Drug costs	21,842.68 €	41,842.96 €	117,578.96 €	103,641.74 €
Of which PI	- €	23,606.10 €	113,152.56 €	102,535.14 €
Of which (P)R	21,842.68 €	18,236.86 €	4,426.40 €	1,106.60 €
Pre-treatment evaluation	170.99 €	85.00 €	85.00 €	85.00 €
Monitoring costs	1,287.97 €	1,207.16 €	1,084.00 €	878.97 €
Adverse event cost	450.07 €	923.49 €	7.00 €	162.53 €
SVR, F0-F2	50.75 €	66.06 €	46.86 €	94.11 €
SVR, F3	25.34 €	13.18 €	15.16 €	28.64 €
SVR, F4	63.40 €	27.77 €	31.94 €	60.34 €
F0-F2	703.42 €	491.51 €	778.50 €	72.15 €
F3	2,881.21 €	1,109.77 €	1,268.17 €	185.75 €
F4	1,258.16 €	1,082.28 €	1,105.84 €	187.26 €
DCC	2,963.85 €	2,595.90 €	2,614.33 €	450.92 €
HCC	1,459.78 €	1,307.52 €	1,348.52 €	588.77 €
LT	1,133.50 €	987.14 €	998.37 €	219.54 €
pLT	1,067.81 €	939.97 €	929.03 €	213.03 €

*PR* PegInterferon and Ribavirin, *SOF* Sofosbuvir, *SMV* Simeprevir, *SVR* sustained virologic response, *PI* protease inhibitor, fibrosis states F0-F4 according to METAVIR, *DCC* decompensated cirrhosis, *HCC* hepatocellular carcinoma, *LT*, first year after liver transplant, *pLT* further years after liver transplant

Costs for IFN-free regimens are dominated by DAA costs. For SOF/SMV, DAA costs are € 102,535.14. Along with a much improved SVR rate, SOF/SMV saves € 5,152.67 in costs for long-term complications DCC, HCC and LT.

The maximum cost defined by the efficiency frontier can be met for SOF/SMV by negotiating a rebate of 6.9 % for SOF (lowering SOF’s 28-unit price from € 18,858.76 to € 17,556.86) or by negotiating a rebate of 8.5 % for SMV (lowering SMV’s 28-unit price from € 15,319.62 to € 14,017.72).

**Fig. 3:**
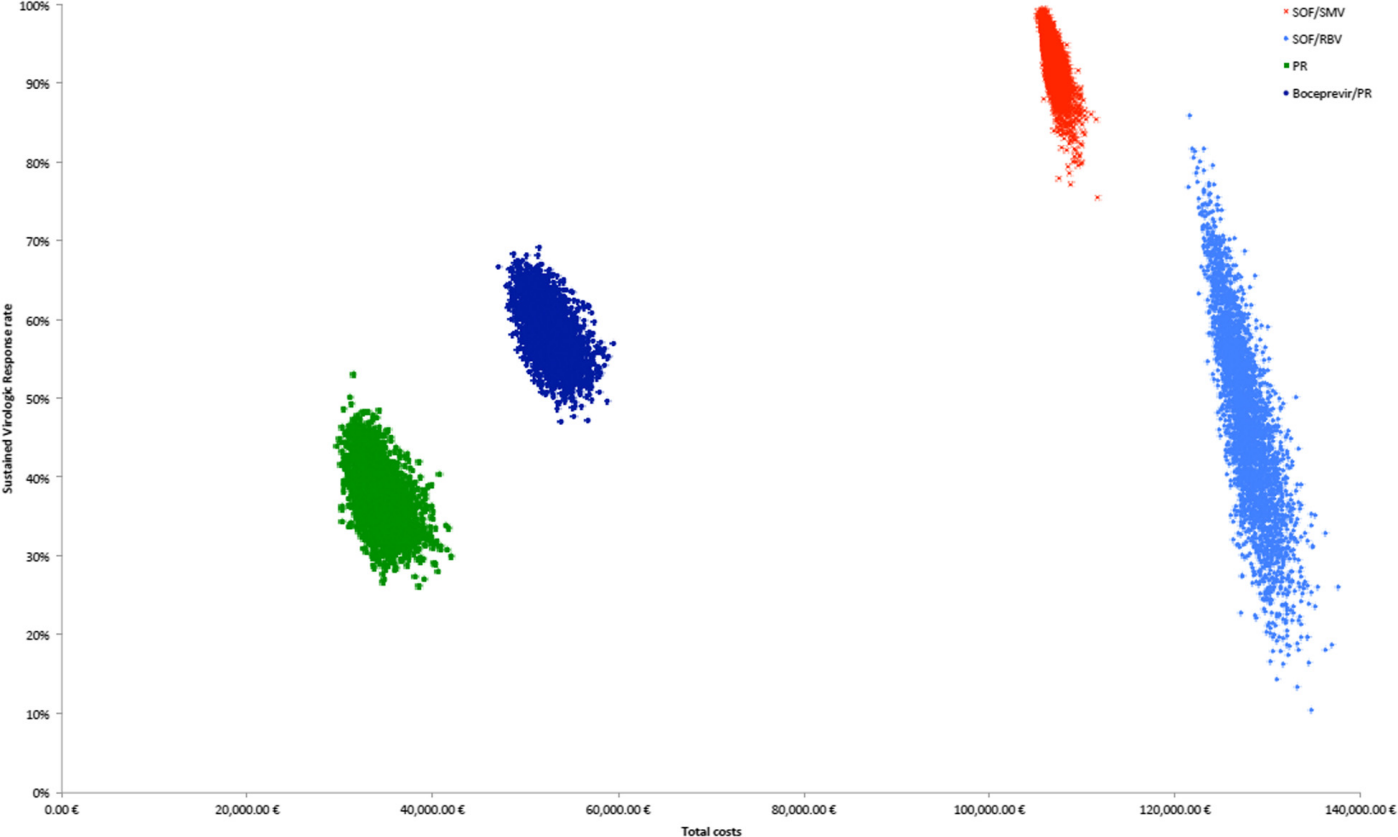
Probabilistic sensitivity analysis. Probabilistic Sensitivity Analysis shows 3000 random draws for each of the four therapies. Out of the 3000 draws, SOF/SMV would be above or on the efficiency frontier in 10.0 % of the simulations, while SOF/RBV would never meet the requirement

Our probabilistic sensitivity analysis shows that even without any rebates, SOF/SMV meets the efficiency frontier requirement in 10.0 % of the cases, while SOF/RBV is never above or on the efficiency frontier at current price levels (Fig. 3).

## Discussion

The cost-effectiveness of HCV therapy has been subject to extensive research in Germany and served as a proof of concept to establish the efficiency frontier method [27, 28]. Since the introduction of DAAs, cost-effectiveness analysis of drugs became more important because antiviral treatment became a major cost component in overall HCV treatment costs [29]. Other studies used incremental life years or QALYs as a measure of benefit for DAAs [20, 30, 31].

For the first time in the history of the treatment of chronic HCV, therapy regimens without the requirement for IFN are available. Within few months, two different regimens received conditional approval for the European markets. SOF/RBV and SOF/SMV are the first opportunity for all-oral treatment without patients having to face the side effects caused by IFN therapy. Both regimens are more expensive than first generation DAAs but save long-term costs for HCV complications.

As our application of the efficiency frontier shows, it is a suitable method to plot both SVR rates and associated long-term costs in one diagram. Due to the decisive nature of SVR rates in the measurement of HCV therapy success, no weighting algorithm is needed to aggregate multiple parameters. Since the introduction of pricing negotiations in 2011, no cost-effectiveness analysis has been used yet to renegotiate an initial rebate agreement. Our analysis shows that the efficiency frontier method is suitable to serve such a purpose for DAAs in HCV therapy.

Our model allows to report the immediate short-term effectiveness of the available treatment regimens, while calculating the impact of the treatment success on long-term complications such as DCC, HCC and LT.

Application of the efficiency frontier approach shows that SOF/SMV therapy almost meets the required threshold, even though DAA prices have not been negotiated yet. While our model incorporates long-term benefits in terms of prevented long-term complications, it does not incorporate the cost-savings associated with preventing disease transmissions by untreated HCV patients.

Our study is limited by the lack of head-to-head trials for direct comparison of SOF/SMV and SOF/RBV treatment regimens. Our application of IQWIG’s approach can be criticized for defining an efficiency frontier with treatment regimens, which are not IFN-free and lack a decisive benefit of SOF/RBV and SOF/SMV. However, our results deliver data for the value of additional SVR percentage points gained in the German SHI system.

Our analysis confirms the findings of a recent study conducted for the US context [31]. SOF/SMV is more effective than SOF/RBV and saves costs both in Germany and in the USA.

The new DAAs are more expensive but our modeling approach shows that they prevent expensive long-term complications more effectively than first generation DAAs. While immediate drug costs are high, each case of prevented LT equals savings of more than € 150,000 in the German SHI system.

Our analysis has shown that SOF/SMV is the most cost-effective IFN-free therapy regimen at the moment. Costs are lower for 12 weeks of SOF/SMV than for 24 weeks of SOF/RBV, both in terms of drug costs for the antiviral therapy and in terms of prevented long-term complications.

SOF/SMV requires therapy for only 12 weeks, which could contribute to better patient compliance. The SVR rate of more than 93 % is unprecedented for all-oral, IFN-free HCV therapy and overall costs need to be lowered by less than 4 % to stay within the requirements of the efficiency frontier.

## Conclusions

The increase in clinical effectiveness by new DAA approvals is unprecedented in HCV therapy. Contrary to previous incremental improvements, recently approved DAAs SOF and SMV represent a jump in clinical effectiveness. Health economic methods are required to put the jumps in clinical effectiveness and the costs associated with the new therapies into perspective. Decision makers in many jurisdictions need suitable tools for cost-effectiveness analysis of the first IFN-free therapies in order to make reimbursement decisions.

Our analysis has shown that IQWIG's efficiency frontier approach is a suitable method to evaluate recent DAA approvals SOF and SMV for IFN-free therapy. In addition to higher sustained virologic response rates, the evaluated therapies save long-term costs by preventing complications such as liver cirrhosis, hepatocellular carcinoma and ultimately liver transplants, thereby offsetting part of higher drug costs. IQWIG's efficiency frontier approach can also be applied to further new approvals for the treatment of HCV, which are expected in the near future.

## Abbreviations

HCV: Hepatitis C virus
DCC: Decompensated cirrhosis
HCC: Hepatocellular carcinoma
LT: Liver transplant
DAA: Direct-acting antiviral agent
SVR: Sustained virologic response
IFN: PegInterferon
RBV: Ribavirin
PR: Combination of IFN and RBV
PI: Protease inhibitor
DGVS: German Society for Gastroenterology, Digestive and Metabolic Diseases
SOF: Sofosbuvir
SMV: Simeprevir
SHI: Statutory Health Insurance
GBA: Federal Joint Committee
IQWIG: Institute for Quality and Efficiency in Health Care
QALY: Quality-adjusted life year
